# Psychographic variables, tourist behaviour and vandalism in the South-East Asian tourism sector

**DOI:** 10.1371/journal.pone.0252195

**Published:** 2021-06-03

**Authors:** Abhishek Bhati

**Affiliations:** Business School, James Cook University Singapore, Singapore, Singapore; University of Bologna, ITALY

## Abstract

This study examines multi-stakeholders’ perceptions of vandalism at tourist attractions in two Asian cities: Bangkok and Singapore. It provides an opportunity to explore the differences and similarities in stakeholder attitudes towards attraction management and reveals desired levels of participation of community in managing vandalism in tourism. This mixed method research employs community survey and interviews of site managers and government officials as its main data collection approach. It also offers an innovative approach to data analysis using the severity and optimist/pessimist psychographic variables coupled with quantitative analytical techniques. The results reveal complex relationship between psychographic profiles, future and current time dimensions, and location. In conclusion, the study offers several recommendations to city managers and policymakers on methods of vandalism control. It also highlights the importance of cultural context and its influence on community involvement. While this study is limited to tourism attractions, it provides a solid foundation for future research, one that can extend into urban planning and public policy design.

## Introduction

This study explores the perceptions and attitudes of key stakeholders and local community to vandalism in tourism sector. The key stakeholders are broadly identified as attraction management, i.e., site managers and local government officials responsible for policies and procedures that inform and guide visitor behaviour. The identified key stakeholders include individuals directly involved in site management. Direct involvement incorporates but is not limited to activities such as repair, maintenance, development of the infrastructure and civic amenities within and around the visitor attractions. In this study, local communities are identified as both residents and businesses within a one-kilometre radius of the tourist attractions being investigated. Tosun [1] states that local community can influence the development of tourist attractions and enable their success or failure within tourism industry. As such, local attitudes need to be considered in order to develop successful strategies to control property damage.

Vandalism is a rather broad term encompassing a wide range of activities [2]. There is not one widely agreed upon definition of vandalism [3]. In fact, there is an argument that a general definition of vandalism is of limited use to researchers and a tailored definition might be more suitable [4]. In this context, vandalism is defined as acts of property damage that include graffiti, carving, litter, breakage, defacing, public property damage, private property damage, and environmental damage. This definition is a distillation of the responses, observations and findings offered by Martin [5], Weinmayr [6], Cohen [7], Zeisel [8], Goldstein [3], and Bhati and Pearce [4,9]. Here, public property damage is identified as damage to commercial facilities owned by local authorities (e.g., City Council), while private property damage is damage to private (privately owned) facilities.

While investigating the complex relationship between positive psychology and tourist behaviour, Pearce [10] identified the need to study optimism and the underlying dimensions of tourists’ satisfaction in order to better assess the emotions of visitors during their experience. A more recent study identified several factors—namely fear, over-reaction and pessimism—that can influence tourists’ perception of risk, thereby changing their behaviour and attitude towards a location [11]. Bae and Chang [12] and Bhati et al. [13] highlight different psychological and perceived risks as key factors in affecting travel behaviour, particularly future travel intentions. Drawing on the above research, this study employs optimist/pessimist psychographic variables and severity index to better assess the emotions of people that frequently interact with tourist attractions. Even though these interactions do not involve tourists but rather community and stakeholders, the study of the underlying dimensions of satisfaction even among multi-stakeholders may contribute to the understanding of attitudes towards a tourist attraction.

There are few papers that investigate and analyse multi-stakeholders’ attitudes towards property damage in Southeast Asia, specifically such acts of vandalism as graffiti, carving, litter, breakage, defacing, public property damage, private property damage, and environmental damage (from now on: property damage). Kattiyapornpong, Ditta-Apichai, Kanjanasilanon, and Siriyota [14] state that multi-stakeholders play a critical role in sustainable tourism development. Their participation facilitates the mutual benefits of all tourism stakeholders. The effective coordination in the context of multiple challenges faced by the tourism industry requires the involvement and cooperation from multi-stakeholders such as communities, enterprises, supporting industries, public sectors and local communities [15]. As such, there is a need to better understand local leaders’, community and policy makers’ perceptions toward tourism development as they act as the initiators in sustainable tourism planning [14,16]. This study offers an analysis of the stakeholders’ attitudes towards property damage as well as their involvement in addressing property damage issues at visitor attractions in the tropical settings of Bangkok and Singapore. The research objectives can be summarised as follows:

1. Identify distinctive stakeholder groups and investigate their attitudes towards vandalism and its prevention; use psychographic variables to help identify sub-groups within the sample?
2. Compare community attitudes and perceptions towards vandalism and its prevention across two culturally and socially divergent tourism destinations in Asia; identify the relationship between community psychographic profile (optimists and pessimists), time orientation (current and future), and location (Bangkok and Singapore).
3. Explore the effect of the psychographic variables (i.e., severity and optimists/pessimists) and location (Singapore and Bangkok) on the desired level of community involvement.
4. Compare multi-stakeholders’ attitudes and perceptions of different types of property damage and its prevention.

Sound management of tourist attractions must incorporate the joint action of government agencies and the community [17,18]. The study of multi-stakeholders’ attitudes, perspectives, and opinions provides comprehensive information that can reinforce strategies adopted to develop sustainable practices in visitor behaviour management [19]. Noticeable degradation decreases the appeal of attractions which in turn affects tourist experience and satisfaction [20]. Significant vandalism could in time result in a reduction in tourist flows even to popular destinations such as Bangkok and Singapore. Vandalism can compromise the social welfare of the community, industry, and government. Current research places an emphasis on collaborative arrangements, stakeholder analysis, and holistic interpretations to reduce the tensions between the tourism industry, visitors, environment, and the communities [17,21–23].

Literature suggests that demographic descriptors are useful indicators for describing sub-group opinions among stakeholders [24,25]. Psychological profiles coupled with quantitative analytical techniques are also useful in producing classifications of observed and measurable individual and group characteristics. This study goes beyond using single demographic descriptors and single psychological profiles. As its primary research strategy, it employs constructed psychographic profiles. Instead of using a single variable characterizing attitudes, this study employs a priori approach [10]. This approach allows for the definition of individuals using non-obscure labels. Current literature reveals that applied labels such as ’optimists and pessimists’ and ’severity’ are easy to evaluate, and provide a simple, meaningful approach to communicating results [26,27].

This study provides an opportunity to understand the differences and similarities in stakeholder attitudes towards attraction management and reveals desired levels of participation of stakeholders and community in managing vandalism in tourism. It builds on prior research by offering an innovative approach to data analysis using the severity and the optimist/pessimist psychographic variables.

## Methodology

The research presented and reported in this project was conducted within the guidelines for research ethics outlined in the National Statement on Ethics Conduct in Research Involving Humans (1999), the Joint NHRMC/AVCC Statement and Guidelines on Research Practice (1997), the James Cook University Policy on Experimentation Ethics, Standard Practices and Guidelines (2001), and the James Cook University Statement and Guidelines on Research Practices (2001). The research methodology received clearance from the James Cook University Experimentation Ethics Review Committee (Human Ethics Approval Number: H4139).

This study employs a community survey to identify and measure the level of current involvement and the desired levels of participation of communities in property damage management. The questionnaire consisted of 29 questions and four sections: 1) attitude towards property damage; 2) attitude towards actions taken to address property damage; 3) desired level of personal involvement in property damage management; 4) demographic characteristics of respondents. Multi-stage cluster sampling has been used to identify individuals based on their ability to influence property damage, as well as individuals most affected by the outcomes of property damage. While property damage affects not only locals but also the wider community, the investigation is limited to the study of communities who can play a more direct role in attraction monitoring and management. The use of a one-kilometre radius suits the research needs and is a sensible solution when canvassing the public for opinions.

This study is part of the ongoing investigation into vandalism and its effects on tourist perception of two pivotal Asian cities: Bangkok and Singapore. The initial inquiry was launched in 2014 and continues to this day. Despite certain similarities (tropical settings, rapidly developing tourism infrastructure, the ‘Southeast Asian’ context), the socio-cultural background of the two locations differs significantly (more details in section ’Results and discussion’). The investigation focuses on urban tourist attractions: cultural and natural attractions, as well as other man-made attractions. An important step was to ensure comparability of attractions (sites) in Singapore and Bangkok to arrive at comparable data for the analysis. Overall, five parameters of site comparability were outlined in the study:

- Within the city (municipal) limits,
- Accessible by public transport,
- Provide a number of tourist related services,
- Offer opportunity to collect data within the ethics approval guidelines,
- Engagement of multiple interests and decision-making networks.

After a physical audit of 22 sites and further testing for within group and intra-group similarities, eleven sites in Singapore and eleven in Bangkok were chosen for this investigation using a rigorous review framework of nine dimensions (visibility, accessibility, perceived safety, general maintenance, surveillance, general security, stakeholder participation, amenities, evidence of vandalism). The communities—residents and businesses—were ‘clustered’ around the identified attractions. Guided by the clustered sampling approach, a total of 600 randomly selected individuals, 300 in each country, were approached. This resulted in 393 valid responses (response rate of 65%): 168 valid questionnaires in Singapore and 225 in Bangkok. The obtained data were analysed using SPSS (20.0).

During the second stage of the investigation, the study employed semi-structured interviews as the main instrument of data collection. Site managers responsible for the day-to-day management and operations of the attractions as well as key government officials were chosen to participate in the study (Table 1). Convenience sampling has been used as the most effective method of sampling for the purposes of this part of the investigation. The participants are selected based on their availability and willingness to take part in the study. Of the 26 interviews, 14 took place in Bangkok and 12 in Singapore.

**Table 1:** Lists of interviewees in Singapore and Bangkok.

S. No	Location	Organisation	Visitor Attraction	Site Manager/Government
1	Bangkok	Jim Thompson Museum Council	Jim Thompson museum	Site manager
2	Bangkok	Prathumwan Khet District Office	Prathumwan City centre	Government
3	Bangkok	Dusit Zoo Management Office	Dusit Zoo	Site manager
4	Bangkok	Wat Po Administration Council	Wat Po temple	Site manager
5	Bangkok	Khao San Police Station	Khao San Road	Government
6	Bangkok	Tourist Police Bangkok	All attractions in Bangkok	Government
7	Bangkok	Lumpini Park Management Office	Lumpini Park	Site manager
8	Bangkok	Sampontham Khet District Office	Chinatown	Government
9	Bangkok	Siam Paragon Office	Siam Paragon	Site manager
10	Bangkok	Bangkok Tourism Department	All attractions in Bangkok	Government
11	Bangkok	Bangkok Metropolitan Administration	Chao Praya River	Government
12	Bangkok	Tourism Authority of Thailand (tourist guide)	All attractions in Bangkok	Government
13	Bangkok	Siam Park City Management Office	Siam Park City	Site manager
14	Bangkok	Bangkok Parks Administration Council	All attractions in Bangkok	Government
15	Singapore	Wild Wild Wet Management Office	Wild Wild Wet	Site manager
16	Singapore	NTUC club	All attractions in Singapore	Site manager
17	Singapore	National Parks Board	All attractions in Singapore	Government
18	Singapore	Singapore Tourism Board (tourist guide)	All attractions in Singapore	Government
19	Singapore	Gardens by the Bay Management Office	Gardens by the Bay	Site manager
20	Singapore	Singapore Botanical Gardens Management Office	Singapore Botanical Gardens	Site manager
21	Singapore	Sentosa Development Corporation	Sentosa	Government
22	Singapore	National Heritage Board	ACM Museum	Site manager
23	Singapore	Sentosa Rangers Office	Siloso Beach	Site manager
24	Singapore	Singapore Tourism Board (Lifestyle Precinct Division)	Orchard Road	Government
25	Singapore	Singapore Police Force	Chinatown	Government
26	Singapore	Wildlife Reserve Singapore	Singapore Zoo	Site manager

In line with Zavattaro’s [28] recommendation, efforts were made to avoid leading questions and minimise research bias. The interviews followed a common interview protocol with a set of predefined key questions. The obtained data were analysed using Leximancer text analytics software (4.0).

This study utilises the severity and the optimist/pessimist psychographic variables to explore the complex relationship between psychographic profiles (optimists and pessimists), future and current time dimensions, and location. Key among these is a perceived severity index. This measure provides a cumulative score of the community’s perception of property damage.

*To measure severity*, respondents were asked to give their opinion about the severity of various acts of property damage at attraction sites. They were asked to describe each act of property damage using descriptors such as ‘major problem = 3’, ‘minor problem = 2’, or ‘not a problem = 1’. Individuals with a mean score of ≥2 for all acts of property damage were regarded as individuals who believe property damage to be a problem. A perceived severity index ranging from 1 to 3 was constructed as an aggregate of responses—an average score—by each respondent to all eight categories of property damage.

To measure pessimism and optimism, respondents were asked two-paired questions designed to evaluate their perception of property damage at visitor attractions at different points in time. For example,

*Compared to the current level of property damage at the attraction, do you feel the damage one year ago was ’Much less’, ’A little less’, ’Worse’, or ’Not sure’*.

*Compared to the current level of property damage, how do you think the attraction site will change in terms of the property damage in the next two years?*

*Be ‘Much better’, ‘A little better’,’ Worse’, or ‘Not sure’*.

Optimism can be defined as a set of beliefs that leads people to approach the world in an active manner. A core confidence lies in the principle of change, meaning conditions keep improving as life goes on. Contrary, pessimists would assume that the world holds a negative future, and nihilists would claim there is no such thing as "better" [29]. For the purpose of this study, pessimistic respondents are described as individuals who regard historical (past) amount of property damage as less than the current amount of damage; and/or who think that the potential rate of property damage would only escalate. In comparison, ‘optimistic’ respondents described the historic damage as greater than the current property damage rate; they also expected less property damage incidences in the future.

## Results and discussion

### Psychographic variables and community attitudes

This section attempts to answer all research questions outlined at the beginning of this study. Table 2 presents the community perception towards property damage in two different locations.

**Table 2:** Perceived severity index.

Category	Singapore				Bangkok				Overall	
	N	%	Mean	Std. Dev.	N	%	Mean	Std. Dev.	N	%
Not a problem (1)	28	17.2	2.36	.570	10	4.5	2.57	.345	38	9.93
Major or minor problem (≥2)	135	82.8			210	95.5			345	90.07

Table 2 reveals slight differences in the respondents’ perception of the severity of damage depending on the location (Singapore vs Bangkok), with approx. 83% of respondents in Singapore believing property damage to be a problem (severity index ≥2). The results are even higher for Bangkok, with 96% of the respondents considering property damage to be an issue. That being said, analysis revealed no significant difference between perceived severity of property damage between Singapore and Bangkok, with both locations seeing it as a problem.

In Table 3, respondents are categorised as pessimists or optimists depending on their perception of current and future vandalism activities.

**Table 3:** Optimists/Pessimists distribution by location and time orientation.

	Singapore current orientation		Singapore future orientation		Bangkok current orientation		Bangkok future orientation	
	N	%	N	%	N	%	N	%
Pessimists	74	**67.27**	30	23.62	116	**75.82**	30	17.34
Optimists	36	32.73	97	**76.38**	37	24.18	143	**82.66**
Total	110	100	127	100	153	100	173	100

As seen above, over 67% of Singapore respondents maintain a pessimistic view of the current state of affairs at identified attractions in regards to property damage. Bangkok respondents are nearly as pessimistic with over 75% assuming a negative outlook towards current situation. That being said, both locations seem to be optimistic in regard to the future: 76% have a hopeful outlook in Singapore and nearly 83% in Bangkok. A quick cross-tabulation analysis revealed that current ’optimists’ tend to remain optimistic about the future and, importantly, current ’pessimists’ also tend to have a more positive outlook, with few people expecting the vandalism situation to worsen in the future.

Table 4 explores the relationship between the ‘optimist/pessimist’ psychographic variables and the location.

**Table 4:** A comparison of optimists and pessimists by location.

		Singapore		Bangkok		Chi-square test			
		N	%	N	%	Value	df	Sig.	N
**Overall Attitudes**	Optimists	114	82	167	85.6				
	Pessimists	25	18	28	14.4				
	Total	139	100	195	100	0.8	1	0.371	334

The results of the above Chi-square test of the overall positive and negative attitudes indicate no significant difference between locations (Chi-square = 0.80, df:1, p = .37). The cumulative data reveal that a large percentage of respondents (over 80%) maintain an optimistic view regardless of their location. These findings suggest not only a somewhat optimistic orientation in both locations, but also that community perception may change over a period of time. This implies that an appropriate set of strategies could be instrumental in swaying public sentiments over time.

Additional tests were performed to analyse current and future attitudes towards vandalism. The results of the Chi-square test showed that there is a significant relationship between optimists’/pessimists’ current and future views in both locations (Singapore Chi-square = 36.19, df: 4, p< .001 and Bangkok Chi-square = 63.13, df: 4, p< .001). In both locations, there is a correlation between current and future attitudes towards vandalism. Results suggest that the future for pessimists seems to have a slightly more hopeful, positive outlook, with respondents in both locations somewhat expecting the vandalism levels to drop. That being said, current optimists tend to be more hesitant in their attitudes towards the future.

The above results draw attention to the importance of engaging community and stakeholders as a way of influencing and modifying public perception. Azadi et al. [30] used a multi-stakeholder involvement approach in evaluation of the urban green space performance and found that state and society have an influential role in place performance. Similarly, this study argues that key stakeholders and community should play a role in tourist location management, particularly when it comes to vandalism control at tourist sites. It has been noted that community engagement produces long-term effects in curbing vandalism [31–33]. Timely interventions and knowledge of preventative measures can promote public’s appreciation of intervention strategies implemented by key stakeholders. Often, community involvement will raise awareness of the need for interventions and may even facilitate necessary timely interventions to reduce property damage [9].

Community involvement in different kinds of site related activities and strategies can range from individual participation to group actions. Participation of individuals and groups can influence and encourage others to join the endeavour, increasing the critical mass needed to effect change. Knowledge of individual preferences is key in encouraging the involvement of community members [29]. Table 5 provides an insight into the desired involvement levels and desired roles distribution in relation to site management activities. The respondents were asked to rate their agreement using Likert scale, where 1 indicates strong disagreement and 5 –strong agreement with a statement.

**Table 5:** Independent sample t-test results: Desired involvement and roles description.

Roles	Overall mean	Singapore	Bangkok	Mean	p–value
		Mean (SD)	Mean (SD)	Difference	
Assist in site management	3.69 (.87)	3.75 (.81)	3.65 (.91)	0.1	p = .01
Involve in decision making	3.81 (.80)	3.7 (.83)	3.9 (.77)	-0.2	p = .04
Give feedback	3.92 (.80)	3.8 (.76)	4.01 (.80)	-0.21	p = .009
Support initiatives	3.91 (.84)	3.69 (.83)	4.06 (.81)	-0.37	p < .001
Participate in reducing damage	3.72 (.88)	3.6 (.89)	3.81 (.87)	-0.21	p = .02
Need local authorities to assist	4.01 (.84)	3.69 (.83)	4.25 (.76)	-0.56	p < .001
Involvement in community action	3.93 (.93)	3.55 (.89)	4.2 (.86)	-0.65	p < .001
Help site management	3.78 (.88)	3.53 (.89)	3.96 (.82)	-0.43	p = .009

Singapore respondents indicate that ‘giving feedback’, ‘site management assistance’ and ‘involvement in decision making’ are of higher priority compared to the rest of the activities. In Bangkok, the respondents require a more active participation of local authorities as well as community participation, particularly in regard to community actions and local support initiatives. Giving feedback also seems to be an important activity for Bangkok residents. Average mean values for both locations reveal that Bangkok respondents see community participation as more desirable or necessary compared to Singapore respondents who tend to be slightly more neutral. It is important to note that there are many types of behaviours and activities that can elicit a response. The questionnaires can offer only limited number of activities and the choice of activities is susceptible to researchers’ bias. This must be acknowledged as a study limitation.

The attitude towards ‘supporting initiatives’ seems to differ between the two locations. With Bangkok respondents, this particular option is considerably more popular (diff = 0.37, p < 0.001). Bangkok respondents also show a stronger preference towards involvement in community actions (diff = 0.65, p < 0.001) and require more local authority assistance (diff = 0.56, p < 0.001) compared to Singapore. While it is hard to definitively say why these differences occur, one could speculate that cultural differences between the respondents may have some influence over the results. Perhaps, Singaporeans more actively rely on the on-site management interventions rather than community interventions believing the former to be more effective, which could also explain their somewhat low enthusiasm for helping the site managers. This can be partially explained by the highly efficient manner of Singaporean public service sector [34] and well-developed law enforcement efforts [35]. Bangkok, on the other hand, generally shows a preference for community involvement [36,37]. Thailand’s public service is less efficient, so Bangkok community may see the need to involve both local authorities and community to facilitate positive change [38]. Looking into the future, it is advisable that urban planners and tourism officials take into consideration these peculiarities, creating more accountable structures and enabling stronger community links for the implementation of effective preventative strategies at tourist sites, specifically in environments where public participation is a strong factor in development initiatives.

Additional independent sample t-test was conducted to further explore whether pessimist/optimist worldviews affect participation levels. The results were not statistically significant, with the optimists and pessimists showing similar amount of desired involvement. Similarly, variable ‘severity’ did not have a significant effect on the participants’ preferred level of involvement. As such, while property damage is considered a problem it does not necessarily translate into an increased desire for involvement.

The relatively high mean scores in Bangkok sample suggest a higher necessity for the community to participate in initiatives designed to address property damage. One could speculate that local authorities in Bangkok are unable to provide adequate guardianship, surveillance, and maintenance of their visitor attractions, so the community inadvertently puts the issue into spotlight by showing an increased desire for involvement. The instruments used to measure desired levels of community involvement can be an indirect way of measuring stakeholder ‘competency’ or even efficiency, where a higher need for community involvement indicates some issues within the internal management systems.

### Psychographic variables and stakeholder attitudes

Data from semi-structured interviews were analysed to explore the attitudes of different stakeholder groups, Bangkok and Singapore government officers (BGOs and SGOs) as well as Bangkok and Singapore site managers (BSMs and SSMs), towards property damage. Here, the government officers represent local government officials responsible for policies and procedures that support sites and tourism development, and the site managers represent people involved in activities such as repair, maintenance, development of the infrastructure and civic amenities within and around the visitor attractions. The interview responses were analysed using Leximancer and thematic analysis. The purpose of this investigation was to understand how different stakeholder groups perceive different levels of property damage and whether their attitude changes over time.

Table 6 provides basic descriptive statistics of stakeholder responses in regard to levels of vandalism today compared to the past, and in future compared to today.

**Table 6:** Stakeholder attitudes towards property damage.

Stakeholder group	Response	Lesser today compared to past		Lesser in future compared to today	
		N	%	N	%
SGOs	Yes	3	60%	3	60%
	No	2	40%	2	40%
SSMs	Yes	3	43%	5	71%
	No	4	57%	2	29%
BGOs	Yes	1	14%	3	43%
	No	6	86%	4	57%
BSMs	Yes	4	57%	3	43%
	No	3	43%	4	57%
Government officers	Yes	4	33%	6	50%
	No	8	67%	6	50%
Site managers	Yes	7	50%	8	57%
	No	7	50%	6	43%

Over half of SGOs (60%) suppose that the level of property damage has reduced over time and is lesser today compared to the past. They see the same trend going forward, with 60% reporting a potential reduction of damage in the future. The majority of BGOs (86%) think that the level of property damage did no decrease and would probably not decrease in the future (57%), though 43% remain hopeful. BSMs, however, see an improvement, with 57% reporting less damage today compared to the past, yet looking ahead they somewhat lose their optimism by suggesting that things might not necessarily improve in the future (57%). Conversely, 57% of SSMs do not see an improvement today compared to the past, yet are generally quite optimistic about the future, with 71% expecting less property damage in the future. Overall, site managers tended to be neither strongly optimistic nor pessimistic, yet government officers were somewhat more pessimistic about current affairs.

A further thematic analysis revealed that SGOs, previously identified as optimists, were likely to use themes related to ‘social responsibility’ with linked nodes such as ’community’, ’society,’ and ‘educated’ but also ’enforcement,’ ’example,’ and ’maintenance’. These notions support the assumptions voiced in previous section, where high levels of community involvement did not seem to be a necessity in Singapore. High levels of site maintenance and law enforcement, as well as educated society and efficient government contribute to a more optimistic outlook. As such, policy makers should not expect high levels of community participation in Singapore (especially since community does not see it as a necessity) and should not spend too much funding on initiatives that try to boost that behaviour. Typical comments from this group included:

*I have been in this industry for the last 33 years. The general environment in Singapore has improved a lot over the years*.

*Singapore has strict laws on vandalism, and in general, there is not much happening. Tight enforcement of fines and behavioural advices has reduced incidence of vandalism*.

Among the quite pessimistic BGO group, analysis revealed nodes such as ’damage’, ’law’, ’awareness’ as well as ’new markets,’ ’tourists,’ and ’activities’. The social themes (e.g., social responsibility, community support) seemed to be weaker in this group. Instead, the officials tended to concentrate on various activities and significant number of tourists, many coming from emerging markets—which seemed to add an element of chaos and sense of disorder. Other problems were associated with lax law enforcement and low awareness within the society, as well as poor behaviour of certain stakeholders, community members and tourists. For example, one official noted that:

*Weak enforcement of law and policies is the main reason for increasing levels of vandalism. Lack of awareness and education of people also result in poor behaviour*.

Another official stated that:

*People and street vendors do not follow the law. They do not feel the responsibility to keep their city damage-free. We need more strict rules and regulations to protect our property*.

The SSM group seemed to rely on conscientious maintenance to take care of property damage, with the analysis revealing themes such as consistency and efficiency. This might explain their overall optimistic future outlook. The following statement made by an attraction website manager is a good example:

*Damage to property does not happen often on Sentosa, and I do not see it as a serious problem. Most of the damage such as litter and graffiti can be removed, so I will not consider it a serious problem*.

*It [vandalism] has not aggravated to a worst case or worst situation. It’s quite constant. Not sure of the actual reasons. People are more prone to silly behaviour*.

Similar comments from other SSMs suggest that while property damage is recognised as a problem, the current approach—namely repair, maintenance, and restoration—ensures that the sites are efficiently run and retain an attractive appearance. Typical comments include,

*It’s [vandalism] more of a nuisance than a problem. It has not caused serious damage, but it is part and parcel of a public place management*.

The final stakeholder group, BSMs, suggest that the situation is slowly improving with the help of new security measures (linked nodes: guards, technology, monitoring) and attitudinal change within the society (linked nodes: better values, education). Typical comments made by site managers in Bangkok include:

*People are more civilised as compared to the past. With higher education and better values, property damage has reduced over the years*.

*Because of the technology in the form of CCTV, better-trained guards will be able to provide better monitoring and good management*.

Yet for BSMs the future still holds a lot of uncertainty, with poorly planned and uncontrolled tourism growth being part of the problem. According to a site manager,

*Increasing the visitors will put pressure on the system*. *It will also increase damage to natural property*.

Another site manager expressed his pessimism by saying,

*Most of the current problem is the destruction of heritage property and public property damage*, *which is considered to be a significant problem*. *There is lack or education*, *awareness*, *and publicity of property damage*.

The results suggest that there is a lack of public awareness that underlies the usual pattern of property damage in Bangkok. The results from both Bangkok groups suggest that lack of awareness and public education need to be addressed and woven into narrative structures (e.g., public awareness campaigns, advocacy programmes, community education) that help fight vandalism in tourism. The results also suggest that in Singapore a more optimistic future outlook is often linked to social responsibility coupled with efficient site maintenance and economic individualism.

## Conclusion

This descriptive study presents an innovative approach to data analysis. It employs specific psychographic variables such as perceived severity index and the optimist/pessimist framework in an attempt to evaluate attitudes towards property damage at visitor attractions. It proves that a psychographic variable approach is quite effective at discerning community-based perceptions and needs regarding vandalism control and property management. While this study is limited to tourism attractions, it provides a solid foundation for future research, one that can extend into urban planning and public policy design.

The findings clearly suggest that vandalism is a problem in both locations, but the underlying reasons for property damage are different. While Singapore residents acknowledge a certain consistency within their vandalism levels, they also believe that using proper management instruments and consistent public policies could improve the situation in the long run. The Bangkok residents link their fluctuating vandalism levels to ineffective policies and lack of public education. This study draws attention to the importance of engaging community and stakeholders as a way of controlling vandalism in communities were government regulations and enforcement are less than effective. At the same time, the results suggest that enforcing community participation in societies like Singapore with pre-existing high levels of government efficiency might not be effective. Instead, the recommendation is to promote a more individualistic participation with an accent on individual social responsibility.

Another interesting finding suggests that community perceptions can change over time, with optimists switching back to pessimism and pessimists adopting a more positive outlook. This implies that consistency and reinforcement should be an important factor when considering anti-vandalism measures. To retain and grow optimism among community members, any managerial and policy related decisions need to resemble a process, i.e., remain continuous, be predictive, and be intrinsically linked to other decisions.

The investigation shows that there is a difference in desired level of involvement depending on the location and the psychographic profile of the respondents. This finding supports and adds to the work of other researchers who study community roles and public participation. For researchers who study community-based tourism (e.g., [39,40], this study might be a guide for developing new instruments. The knowledge of the nature of the desired participation is helpful in designing intervention strategies as well as getting the community to engage more proactively with initiatives that address property damage.

This study also touched upon some cultural differences and their influence on desired level of involvement. For instance, the results suggest that respondents from Singapore prefer a more personal rather than group involvement, while the Bangkok sample has a preference for group action. In Singapore, for example, the active and effective role of the government has brought on a type of complacency, where the community feels a strong reliance on the local authorities to maintain public spaces, effectively distancing themselves from the problem. The results point to a correlation between levels of government participation and the community’s willingness to engage in initiatives that address property damage. These results support earlier findings from Kret Island, Thailand, where the diffusion theory has been used to diffuse sustainable tourism ideas among destination stakeholders and tourists, noting that this approach can influence the community participation in sustainable development [41].

This study also provides a glimpse into the world of public officials, noting attitudes and responses of key stakeholders such as site managers and government officers towards vandalism. The findings suggest that key stakeholder perceptions and resulting optimism/pessimism are closely linked to locations and cultural backgrounds of communities under investigation. In addition, visitor behaviour and general education levels were perceived as closely linked to property damage at tourism sites. As such, community characteristics should be taken into consideration when planning initiatives that address property damage. The findings also support the existing literature in which psychographic profiles of stakeholder groups are linked to the level of success of campaigns designed to reduce property damage and protect heritage and tourism property values [19,42].

Evidence suggests that people respond differently to developments in their environment depending on whether they have optimistic or pessimistic views [43]. The different attitudes have been shown to produce different patterns of behaviour [44]. The findings in this study show mixed results and perhaps point towards a fluidity in perceptions of both optimists and pessimists. For example, only SGOs retained their optimism for both current and future situations. Even though BGOs were prone to be more pessimistic, their future pessimism was less pronounced compared to current levels of pessimism. It is necessary to note that the key stakeholder sample was relatively small, so these observations require further testing. Additional research is required to test whether these findings can be successfully replicated.

At a conceptual level, the findings seem to support the social representation theory which claims that there can be competing and sometimes contradictory versions of reality existing side by side in the same community [10,45]. The meaning of tourism and the value of tourist sites may vary significantly among different people, even when they belong to the same community [46–48].

In conclusion, the above findings offer a strong foundation for future research into vandalism in tourism. It is possible to extend these finding to other areas of city development, where they can be of use to urban planners and city managers. This study offers several recommendations for policy development that are of particular relevance to urban policymakers and the tourism industry.

## Supporting information

S1 Data (XLSX)
